# Sensory assessment of *Cercospora beticola* sporulation for phenotyping the partial disease resistance of sugar beet genotypes

**DOI:** 10.1186/s13007-019-0521-x

**Published:** 2019-11-16

**Authors:** Erich-Christian Oerke, Marlene Leucker, Ulrike Steiner

**Affiliations:** 1 INRES–Plant Diseases and Crop Protection, Rheinische Friedrich-Wilhelms-Universitaet Bonn, Nussallee 9, 53115 Bonn, Germany; 2 Plant Protection Service, Chamber of Agriculture, Gartenstraße 11, 50765 Cologne, Germany

**Keywords:** Hyperspectral imaging, Cercospora leaf spot, *Beta vulgaris*, Fungal sporulation, Resistance breeding

## Abstract

**Background:**

Due to its high damaging potential, Cercospora leaf spot (CLS) caused by *Cercospora beticola* is a continuous threat to sugar beet production worldwide. Breeding for disease resistance is hampered by the quantitative nature of resistance which may result from differences in penetration, colonization, and sporulation of the pathogen on sugar beet genotypes. In particular, problems in the quantitative assessment of *C. beticola* sporulation have resulted in the common practice to assess field resistance late in the growth period as quantitative resistance parameter. Recently, hyperspectral sensors have shown potential to assess differences in CLS severity. Hyperspectral microscopy was used for the quantification of *C. beticola* sporulation on sugar beet leaves in order to characterize the host plant suitability / resistance of genotypes for decision-making in breeding for CLS resistance.

**Results:**

Assays with attached and detached leaves demonstrated that vital plant tissue is essential for the full potential of genotypic mechanisms of disease resistance and susceptibility. Spectral information (400 to 900 nm, 160 wavebands) of CLSs recorded before and after induction of *C. beticola* sporulation allowed the identification of sporulating leaf spot sub-areas. A supervised classification and quantification of sporulation structures was possible, but the necessity of genotype-specific reference spectra restricts the general applicability of this approach. Fungal sporulation could be quantified independent of the host plant genotype by calculating the area under the difference reflection spectrum from hyperspectral imaging before and with sporulation. The overall relationship between sensor-based and visual quantification of *C. beticola* sporulation on five genotypes differing in CLS resistance was R^2^ = 0.81; count-based differences among genotypes could be reproduced spectrally.

**Conclusions:**

For the first time, hyperspectral imaging was successfully tested for the quantification of sporulation as a fungal activity depending on host plant suitability. The potential of this non-invasive and non-destructive approach for the quantification of fungal sporulation in other host–pathogen systems and for the phenotyping of crop traits complex as sporulation resistance is discussed.

## Background

The imperfect fungus *Cercospora beticola* Sacc. causes Cercospora leaf spot (CLS), the most important leaf disease of sugar beet (*Beta vulgaris*) worldwide [1] and may produce up to six generations within a growing season. A severe disease outbreak in sugar beet fields may cause yield losses up to 50% or more underlining the importance of host plant resistance that leads to a decreased reproduction of the pathogen [2]. Rossi et al. [3] demonstrated that a reduced spore production is an important component in the rate-reducing resistance of sugar beet against CLS. In field trials for promising breeding lines, neighbouring effects may result in a more general inoculum release and will lead to an underestimation of sugar beet genotypes with major effects on the sporulation [4]. Thus, an efficient and reliable tool to quantify the sporulation of *C. beticola* may help to enhance the speed of resistance breeding.

As the resistance of crop plants to major pathogens is often incomplete [5, 6], quantitative differences in pathogen development in crop genotypes have to be assessed. The amount of pathogen (biomass)—compared to the amount on a highly susceptible genotype—is the most objective indicator for the compatibility between host plant and pathogen species, however, hard to measure because the pathogen is not or only partially visible. Instead of that, the direct or indirect effects of the pathogen on the host are evaluated [7]. In general, the quantitative (= partial) resistance of a host genotype is expressed in relative terms compared with the reaction of a well-known standard [8]. For polycyclic pathogens, the total amount of disease (for various genotypes) is often measured in the field by the end of vegetative growth period and includes the sum of various resistance components (field resistance).

The components of quantitative host plant resistance may reduce the chance of infection (penetration resistance), the growth of the pathogen in the plant tissue (colonization resistance), as well as fungal spore production (sporulation resistance) which is crucial for pathogen spread and the build-up of epidemics. It is possible to discern at least three components of quantitative reaction against leaf pathogens; infection frequency, lesion size and sporulation rate per lesion [8]. Associated with lesion size and sporulation rate is the latency period, the period between infection and first spore production. These components tend to be associated with one another in most pathosystems; however, this association varies from very strong to non-existent [9].

Asexually formed conidia of plant pathogens are the main units for reproduction and spreading and are often produced in large amounts during the growing season of the respective host crop. The time and amount of sporulation is crucial especially with regards to the rate of epidemic development of a disease [10]. The cultivation of resistant varieties can decrease the inoculum potential within the field and delays disease epidemics. In case of quantitative disease resistance, the completion of the disease cycle and thus the spore production can be decelerated or prevented [11].

For polycyclic fungal pathogens of plants, the repeated infection cycles include the sequence infection, colonization, sporulation and dispersal to new host plants. Sporulation of the pathogen may be rather independent from the host plant’s susceptibility/resistance to penetration (and tissue colonization) and requires a high degree of nutrient availability from the plant tissue (= host suitability). Especially for pathogens sporulating on necrotic lesions e.g. *C. beticola,* the relationship between disease symptom (and pathogen biomass) and spore production is highly variable. In contrast, the amount of spores of powdery mildew and rust fungi is related to the size of the disease symptom which is formed by the fungal biomass, primarily spores, itself.

For necrotrophic pathogens, the assessment of pathogen sporulation is hampered by the fact, that the sporulation rate (= amount of spores per unit of time) may not be related to fungal biomass (in the host tissue). Furthermore, the production and release of conidia on the plant surface often depends on environmental triggers, e.g. 100% relative humidity (RH) and droplets of free water, respectively. As the spore yield is time-dependent, the quantification of fungal sporulation should be non-destructive. For spores for anemochorous dispersal, spore traps are highly suitable [12]. Although aerial spore traps may be used in the field to predict high propagule densities [13], the release of the long, acicular conidia of *C. beticola* from the conidiophores is favoured by splashing water [14], a fact that facilitates the handling of spore-bearing sugar beet leaves or plants as well as the assessment of sporulation rates. Currently the effectivity and reliability of quantitative disease resistance of sugar beet genotypes has to be tested in the field. An option to assess this epidemiological relevant parameter also under controlled conditions, therefore, would not only benefit the decision-making in genotype selection for resistance (sources), but also a more detailed analysis of the various components of quantitative disease resistances.

As the assessment of quantitative resistance components is laborious—especially measurements of spore production per unit host area, and infection frequency—it has not been advisable for a long period of time to measure one or more of these components, but resistance preferably has been assessed in the field [9].

Hyperspectral imaging (HSI) has been successfully used to quantify plant diseases like *Verticillium* wilt in olive or red leaf blotch of almond [15, 16] and other plant diseases as reviewed by Mahlein [17] and Oerke [18]. In sugar beet, hyperspectral techniques have been demonstrated to be suitable for the detection, identification and quantification of leaf diseases—at least under controlled environmental conditions. Non-imaging spectroradiometers and HSI were not only able to differentiate among Cercospora leaf spot, powdery mildew and rust [19, 20], but Leucker et al*.* [21] also demonstrated the potential of HSI to differentiate among genotype-specific plant reactions to *C. beticola* attack depending on the level of host plant disease resistance. Spectral information at high spatial resolution was suitable for the segmentation of CLS lesions into a reddish margin, a transition area and the centre of the lesions differing in size and composition depending on the phenotype of the leaf spot. As sporulation of the pathogen is limited to the central area of leaf spots [21], the size of this segment is crucial for the assessment of the sporulation capacity of *C. beticola* on sugar beet genotypes.

There are also different sensor-based approaches to quantify CLS in sugar beet ranging from RGB image analyses to multi- and hyperspectral analysis methods [19, 22, 23]. These studies focus on the assessment of disease severity and so far, no approach takes into account the sporulation directly. The dependency of *C. beticola* sporulation on high RH enables the induction of conidiation under controlled conditions. Therefore, a procedure to assess the spore production of *C. beticola* by hyperspectral imaging was developed. Sporulation on genotypes of different resistance level against *C. beticola* was assessed by using a hyperspectral microscope and results of two approaches of data analysis—Spectral Angle Mapper, quantification of spectral differences between spectra without and with pathogen sporulation—were compared to conventional methods for the quantification of fungal sporulation.

## Materials and methods

### Plant material

Five homozygous inbred lines of sugar beet (*Beta vulgaris* L. subsp. *vulgaris* var. *altissima*) were supplied by KWS SAAT SE (Einbeck, Germany) for the experiments; genotypes Bv1 + Q and Bv4_r are (partial) resistant to CLS, Bv6_s and Bv7_s are susceptible to the disease, while Bv2-Q reacts intermediate. Genotype Bv1 + Q carries the alleles of the resistant parent in two quantitative trait loci (QTL), Bv2-Q does not [24]. Resistance of the genotype Bv4_r was developed from a different resistant source. The rating of CLS susceptibility was provided by KWS SAAT SE based on multi-year field trials from the sugar beet breeding program.

Plants were cultivated in the greenhouse as described earlier by Leucker et al. [21]. Plants were used for the experiments at growth stage (GS) BBCH 16 [25].

### Pathogen and inoculation

Inoculum of *Cercospora beticola* Sacc. was harvested from wetted CLS-infected sugar beet leaves incubated at 100% relative humidity (RH) for 48 h. Conidia were washed off with a 0.01% solution of polysorbate 20 (Tween 20, Sigma-Aldrich, Munich, Germany) and a spore suspension with 4 × 10^4^ conidia mL^−1^ was sprayed onto sugar beet leaves. After incubation at 100% RH and 25 °C/ 20 °C day/night temperature for 48 h, plants were put back to 60 ± 10% RH until the induction of *C. beticola* sporulation.

### Measurement of conidia production

Spore production was measured three weeks after inoculation according to Karadimos et al. [26]. Sporulation was induced by incubating diseased plants and detached diseased leaves at 100% RH for 2 days. Leaf disks (Ø 11 mm) with a single lesion each were separately placed into 0.5 ml of tap water with 0.01% polysorbate 20 and vigorously shaken to detach spores from conidiophores. Conidia were counted using a Fuchs-Rosenthal chamber (Brand, Wertheim, Germany). Sixteen to twenty lesions per genotype were analysed and results expressed as the number of conidia per square millimetre diseased leaf area and as the number of conidia per lesion, respectively.

For recording of the hyperspectral difference between lesions with and without conidia, the conidia were removed mechanically from sporulating lesions directly after the first image by using a fine paintbrush.

Sporulating CLS lesions were examined using a Leica MZ16 F stereo microscope (Leica, Wetzlar, Germany) and images were taken with a mounted digital camera KY-F75U (JVC, Yokohama, Japan).

### Hyperspectral measurements and image analysis

Hyperspectral reflection was measured by a hyperspectral microscope (spectral camera PFD V10E, Spectral Imaging Ltd., Oulu, Finland) as previously described by Leucker et al. (2016) and samples were magnified fourfold.

Non-inoculated leaves as control and fully developed CLS lesions on the sugar beet genotypes were measured 19 days post inoculation (dpi) as well as after induction of sporulation (21 dpi). The hyperspectral images were pre-processed according to Leucker et al. [21]. Spectral signatures were manually extracted as average reflectance of 20 lesions per genotype and the area between the two spectral signatures (= area under difference spectrum [AUDS]) recorded before and after incubation was calculated.

The Spectral Angle Mapper (SAM) algorithm [27, 28] was used for supervised classification of spectral information of image pixels. Spectrally unique signatures of pure image components (endmembers; e.g. healthy leaf tissue, CLS margin, centre of leaf spots, conidia, etc.) were stored in a spectral library. SAM calculates the spectral similarity of a pixel of interest and all reference spectra selected in an n-dimensional space depending on the number of spectral bands. Depending on the settings of the algorithm, SAM assigns all pixels to the reference components or leaves them unclassified and illustrates the classification result in a false-colour image.

### Microscopy

A Leica MZ16 F stereomicroscope (Leica Microsystems, Wetzlar, Germany) was used to study the symptom characteristics of the CLS lesions. For light microscopy, hand sections were performed to visualize the melanized conidiophores on the surface of sporulating lesions. Additionally, leaf samples with mature CLS lesions were cleared in saturated chloral hydrate (250 g/100 mL a. dest.) at room temperature for at least 7 days to observe the fungal structures inside the plant tissue using a Leitz DMR photomicroscope (Leica Microsystems, Wetzlar, Germany). Images were saved using the software Discus (Technisches Büro Hilgers, Königswinter, Germany).

Scanning electron microscopic observations were obtained using a Phenom G2 Pure (Phenom-World, Eindhoven, Netherlands). Freshly harvested leaf disks with sporulating CLS lesions were gold coated using an automated sputter coater (MSC 1 T, LOT Quantum Design, Darmstadt, Germany). Images of at least four representative lesions on each genotype were recorded.

### Statistical analysis

Statistical analyses were conducted using SPSS 22.0 (SPSS Inc., Chicago, USA). Data were tested for normal distribution and equity of variances. The means between genotypes were compared using Tukey’s honestly significant difference (HSD) test with a significance level of *P* = 0.05 confidence in order to separate subgroups. Experiments were repeated two times with at least 16 biological replicates per genotype (n ≥ 16).

## Results

### Sporulation of *C. beticola* on attached and detached leaves

**Fig. 1:**
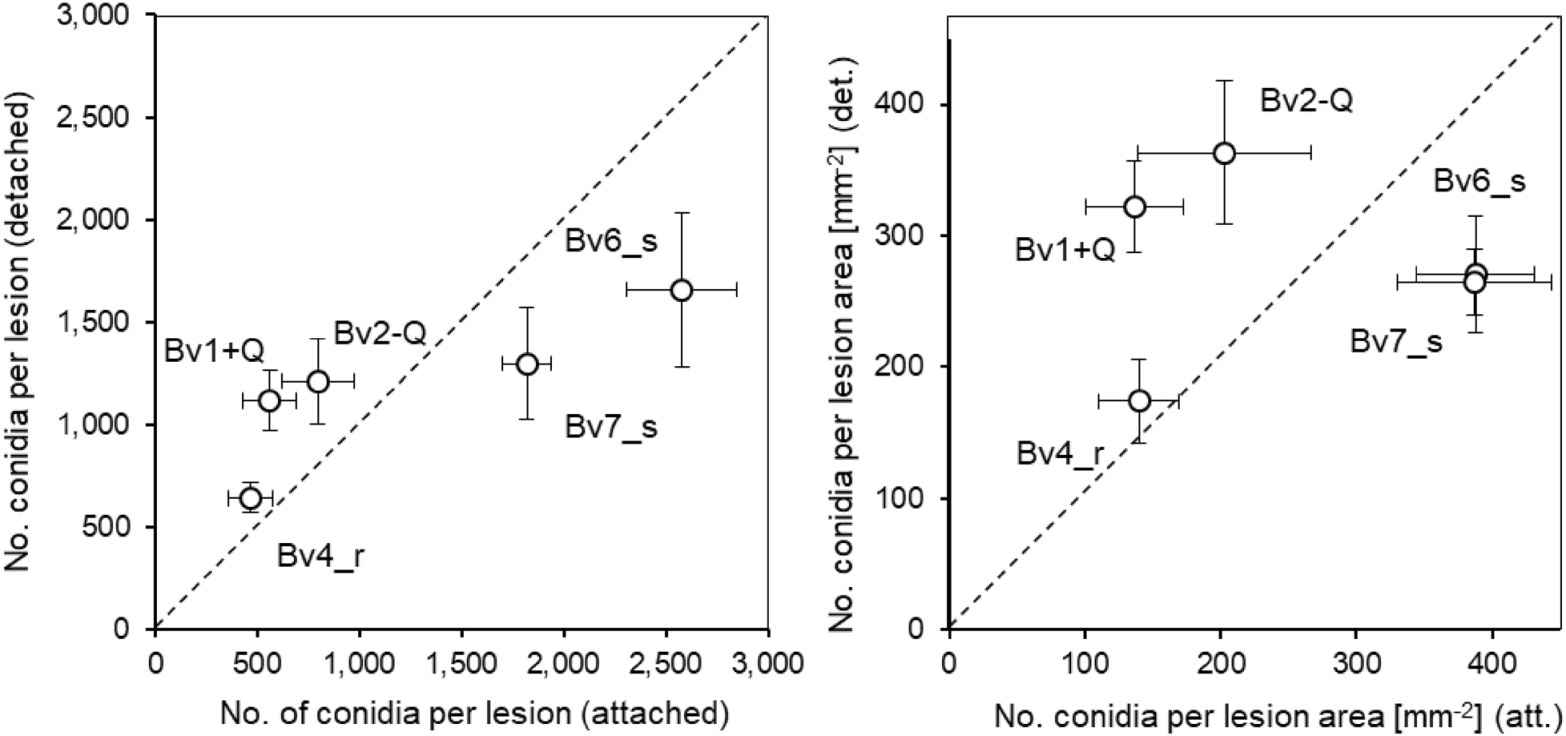
Comparison of conidia production of. *C. beticola* on five sugar beet genotypes differing in CLS resistance on attached and detached leaves during induction of sporulation. Left, production of conidia per lesion; right, production of conidia per area of lesion. Bars represent standard error of the mean

As the retrieval of leaf spots on detached leaves / punched-out leaf disks is much easier than on whole plants with all leaves, the sporulation of *C. beticola* on five genotypes of *B. vulgaris* was tested for CLS lesions on attached and detached leaves. The rate of conidia production within 2 days of 100% RH significantly differed among genotypes and leaf status (Fig. 1). For detached leaves, the sporulation rate on the partially resistant (Bv1 + Q, Bv2-Q, Bv4_r) and highly susceptible genotypes (Bv6_s, Bv7_s) was favoured and reduced, respectively. For both parameters, conidia per lesion and conidia per lesion area the detachment of leaves resulted in a levelling of sporulation among sugar beet genotypes.

**Fig. 2:**
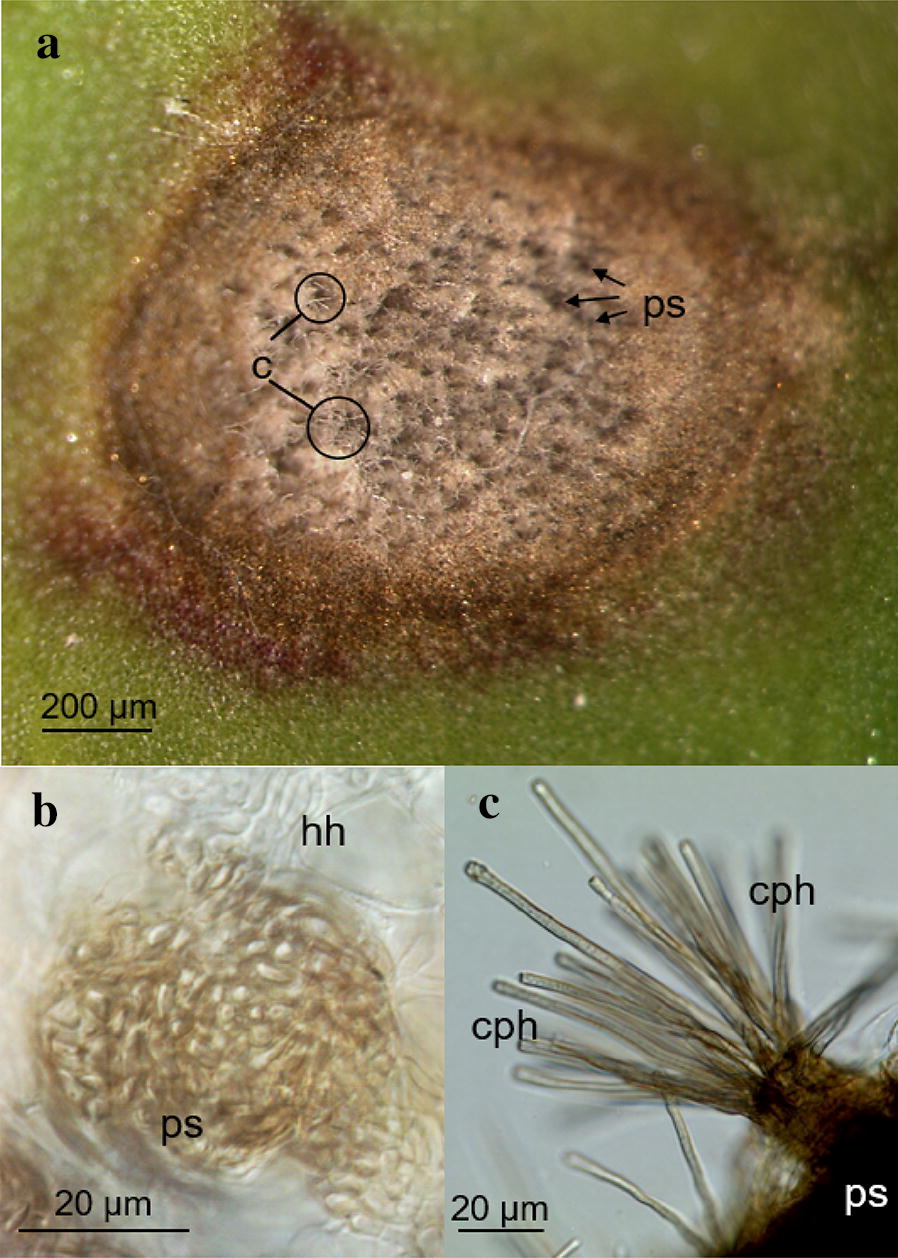
Production of conidia by *Cercospora beticola* on the susceptible sugar beet genotype Bv6_s. **a** Cercospora leaf spot with hyaline conidia (c) produced on dark pseudostromata (ps) in the centre, 21 days past inoculation; **b** hyaline mycelium (hm) in the mesophyll and melanized pseudostroma (ps) in the substomatal cavity; **c** melanized conidiophores (cph) on the leaf surface

**Fig. 3:**
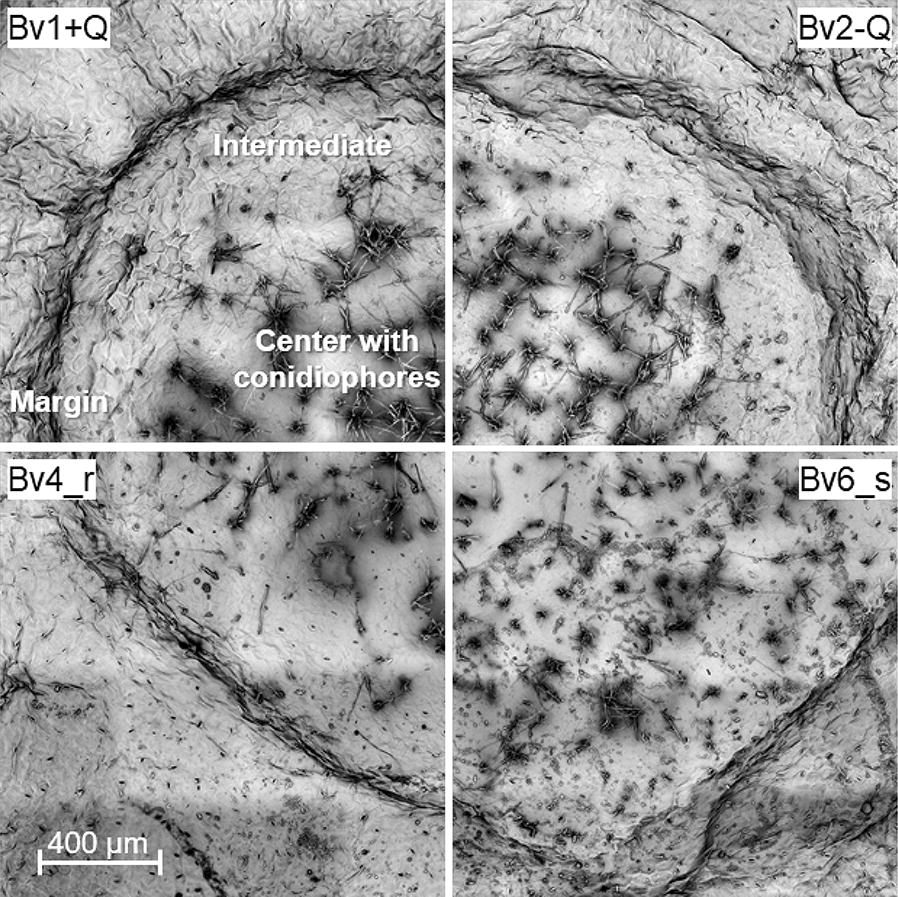
Scanning electron images of sporulating leaf spots of *C. beticola* on four genotypes of *Beta vulgaris* differing in CLS resistance. Leaf spots on partially resistant genotypes (Bv1 + Q, Bv4_r) were smaller and/or had a smaller centre with lower production of conidiophores than those formed on susceptible genotypes (Bv2-Q, Bv6_s)

On susceptible genotypes, *C. beticola* produced hyaline conidia in the centre of CLS symptoms; they arise from dark conidiophores protruding from melanized pseudostromata localized in substomatal cavities. The hyphae colonizing the mesophyll are hyaline (Fig. 2). The host genotype influenced the fungal sporulation on attached leaves in modifying the area and intensity of conidiophore formation (Figs. 3, 6a). The more resistant the host tissue, the smaller was the central part of CLS with conidiophores (and conidia) and the lower was the density of conidia. For the five *B. vulgaris* genotypes, the overall correlation between lesion size and the number of conidia per lesion (R^2^ = 0.05) and the number of conidia per area of lesion (R^2^ = 0.03), respectively, was low (Additional file 1: Fig. S1).

### Spectral assessment of *C. beticola* sporulation in Cercospora leaf spots

**Fig. 4:**
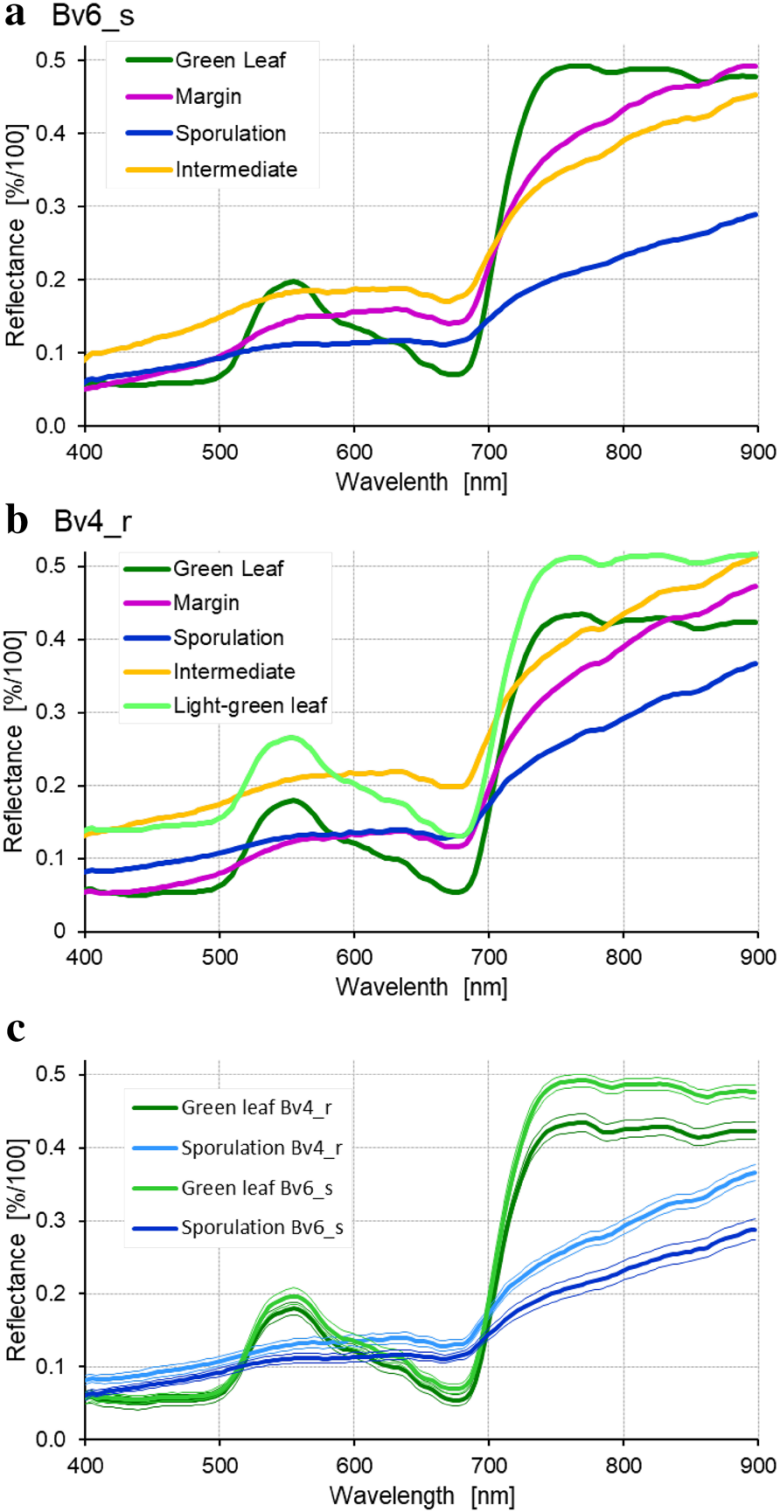
Reference spectra of the components of Cercospora leaf spots on susceptible genotype Bv6_s (**a**) and resistant genotype Bv4_r (**b**). Spectra were extracted from diseased tissue with and without sporulation (margin, intermediate) and healthy green leaf tissue. **c** Comparison of spectra for healthy green leaf tissue and sporulating lesion areas from both sugar beet genotypes, curves represent mean ± SEM (n = 16)

**Fig. 5:**
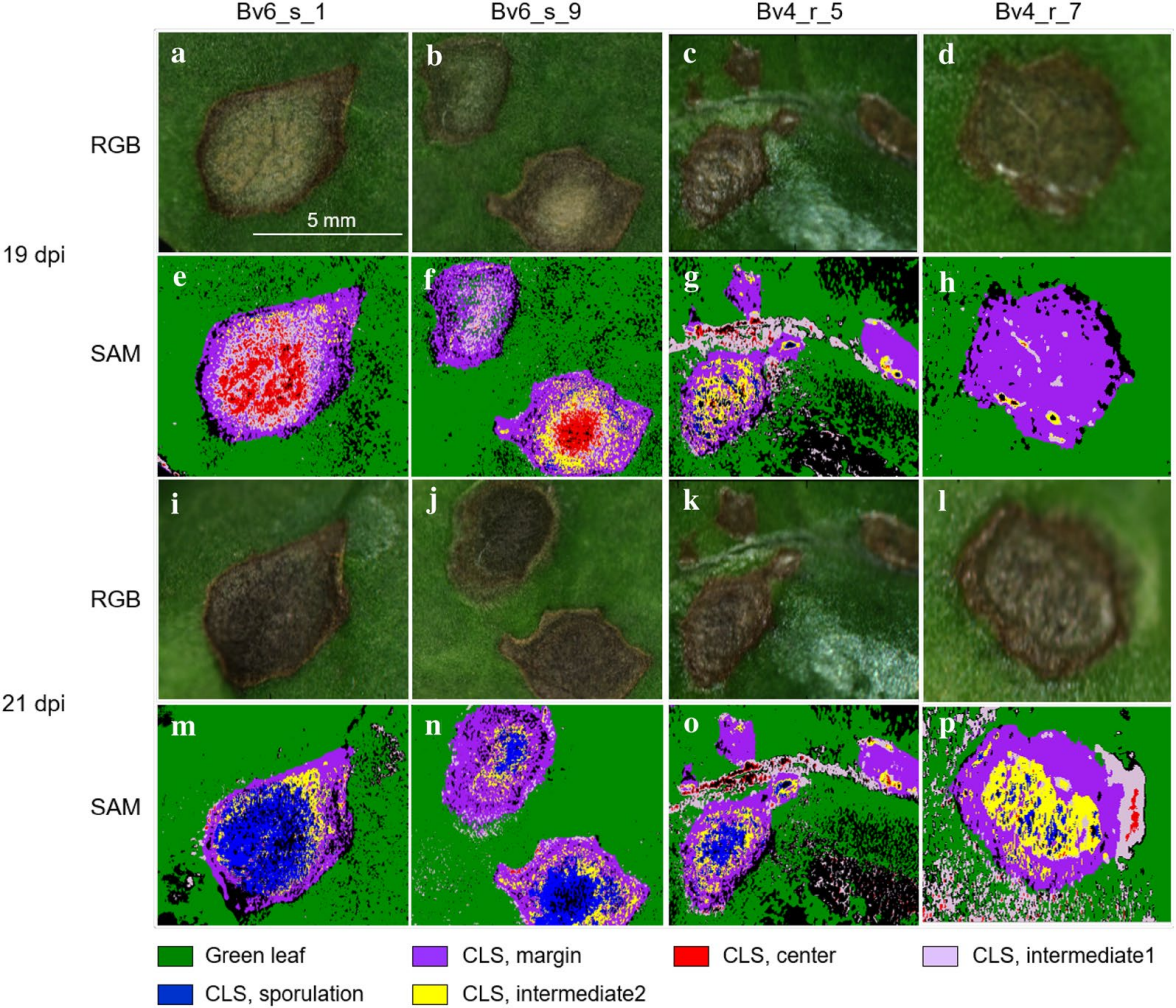
Quantification of Cercospora leaf spot (CLS) components from leaves of two sugar beet genotypes differing in resistance to CLS by supervised classification using the spectral angle mapper (SAM) algorithm. For genotypes Bv6_s (susceptible) and Bv4_r (partially resistant), two leaf areas with lesions were analyzed by recording hyperspectral images which were classified by using reference spectra (given in Fig. 4) in SAM 19 dpi (before sporulation) and 21 dpi (after induction of *C. beticola* sporulation); RGB representations from hyperspectral recording 19 dpi (**a**–**d**) and 21 dpi (**i**–**l**), SAM results 19 dpi (**e**–**h**) and 21 dpi (**m**–**p**). CLS intermediate 1 and CLS intermediate 2 refer to non-sporulating areas before and with sporulation, respectively

**Fig. 6:**
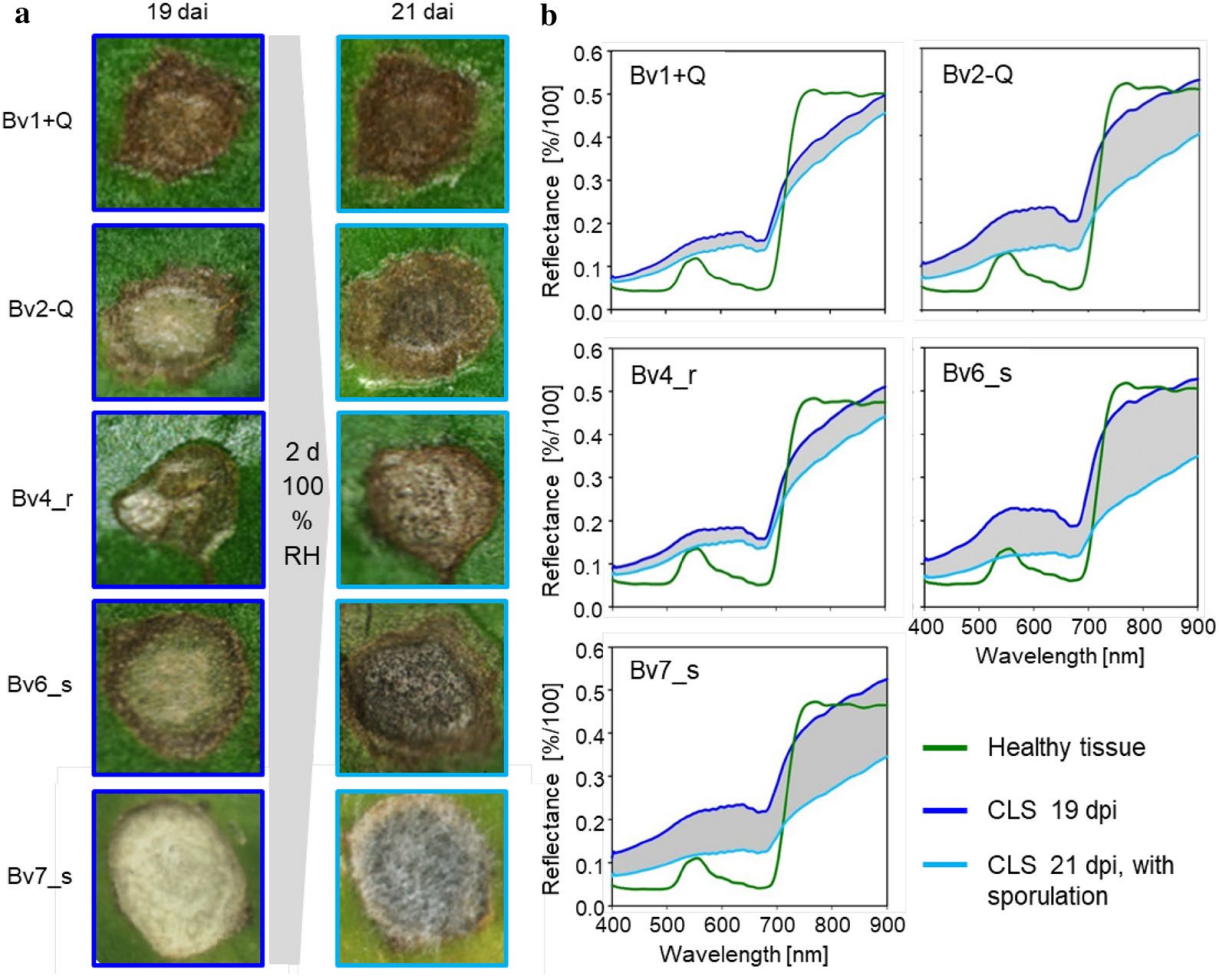
Sporulation of *Cercospora beticola* on sugar beet leaves as quantified by hyperspectral imaging. **a** RGB images of Cercospora leaf spot (CLS) lesions on five sugar beet genotypes differing in CLS resistance before (19 days post inoculation; dpi) and with (21 dpi) sporulation induced by incubation under 100% relative humidity (RH). **b** Spectral signatures of healthy control tissue and CLS lesions (n ≥ 10)

Incubation of plants with typical CLS lesions 19 dpi under 100% RH for 2 d induced the formation of bundles of conidiophores spreading throughout the light centre of the lesions on both sides of the leaves (Figs. 3 and 6a). Conidiophores protruded from darkly pigmented spots (pseudostromata) and bore the acicular, hyaline conidia. The supervised classification of images taken before and after induction of *C. beticola* sporulation was based on the reference spectra of the different CLS lesion parts (Fig. 4). As demonstrated for the susceptible genotype Bv6_s and the resistant Bv4_r, the spectra of lesion margin, lesion centre, intermediate area, and sporulating lesion areas significantly differed from healthy sugar beet tissue as well as from each other (Fig. 4c) and could be used for the classification of lesion pixels. The example of sample Bv6_s_9 in Fig. 5 demonstrated the high potential of hyperspectral imaging for differentiating developmental stages of the leaf spots. The adult leaf spot on the right had the typical zonation margin—intermediate—centre and resulted in heavy sporulation in the central area 21 dpi, whereas the lesion on the left was in an earlier stage and the success of sporulation induction was very limited (Fig. 5). The results of the image classification also allowed a quantification of *C. beticola* sporulation: After induction of sporulation, sporulation was identified on 27.2% and 8.4% of the CLS lesion area for genotypes Bv6_s and Bv4_r, respectively. The repeated measurements on the same leaf spots indicated to an increase of the leaf spot area during sporulation by about 10 to 20% (data not shown).

The spectral angle classification of sporulating areas required the definition of genotype-specific reference spectra, because the spectra, although similar for the various lesion parts on all genotypes significantly differed from *B. vulgaris* genotype to genotype (Fig. 4c). Not only the disease structures on genotypes Bv6_s and Bv4_r had different spectra, moreover, the genotypes of *B. vulgaris* also differed in the spectrum of healthy leaf tissue.

### Effects of *C. beticola* sporulation on the reflectance spectrum of CLS lesions

In order to develop a general, genotype-independent approach of spectral quantification of *C. beticola* sporulation, the average reflectance spectra (from 400 to 900 nm) of non-sporulating CLS lesions on five *B. vulgaris* genotypes were extracted 19 dpi and compared to the reflectance of non-infected tissue (Fig. 6b). Spectral signatures of CLS were characterized by an increased reflectance in the VIS range from 400 to 700 nm and a decrease of the red shoulder. The increase in reflectance on the intermediate genotype Bv2-Q and the susceptible Bv6_s and Bv7_s was higher than on the resistant sugar beet genotypes Bv1 + Q and Bv4_r.

**Fig. 7:**
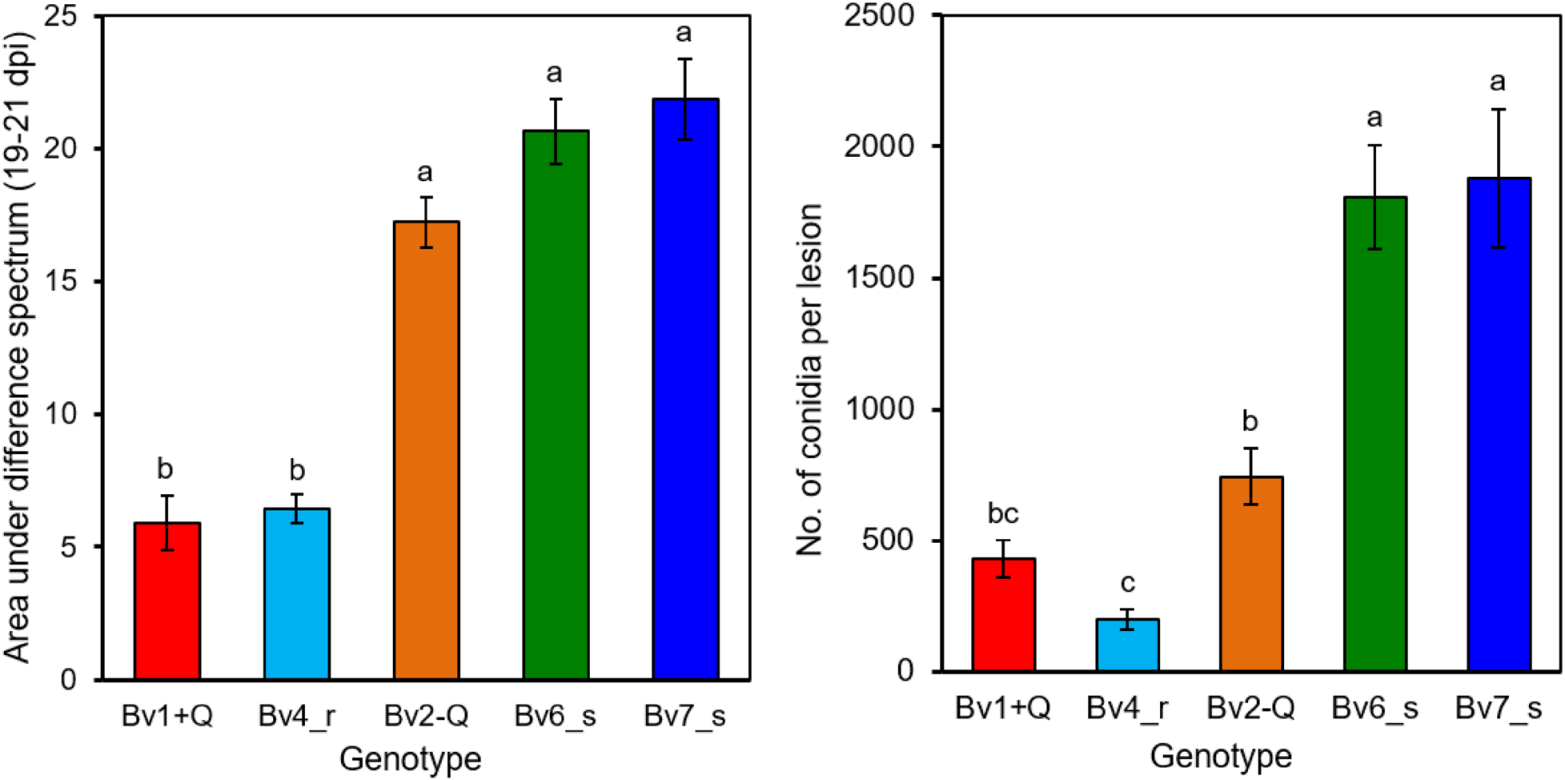
Effect of sugar beet genotype on the sporulation of *C. beticola* as quantified by hyperspectral imaging (left) and counting of conidia (right). The area under the difference spectrum was calculated from spectra taken before sporulation (19 dpi) and with sporulation (21 dpi), respectively. Values with the same letter are not significantly different (Tukey’s honestly significant difference test (p = 0.05, n ≥ 10 [spectra], n = 20 [counts]). Bars indicate standard error of the mean

Incubation under 100% RH for 2 days induced the formation of conidia on conidiophores and led to a strong decrease in reflectance over the whole wavelength spectrum of all genotypes. The spectral differences before and after sporulation—quantified as area under difference spectrum (AUDS)—were greater on susceptible genotypes and so the areas between the two spectral signatures 19 and 21 dpi (indicated as grey area in Fig. 5b) were calculated for each genotype (Fig. 7a). The values were significantly higher for Bv2-Q, Bv6_s and BV7_s than for the resistant genotypes Bv1 + Q and Bv4_r. To compare the spectral differences with the spore production of *C. beticola*, the number of conidia produced per lesion was counted. Over 2000 conidia per lesion were produced on Bv2-Q as well as on Bv6_s and Bv7_s. Compared to that, spore production was significantly lower with on average 500 conidia per lesion on Bv1 + Q and Bv4_r, respectively (Fig. 7b).

**Fig. 8:**
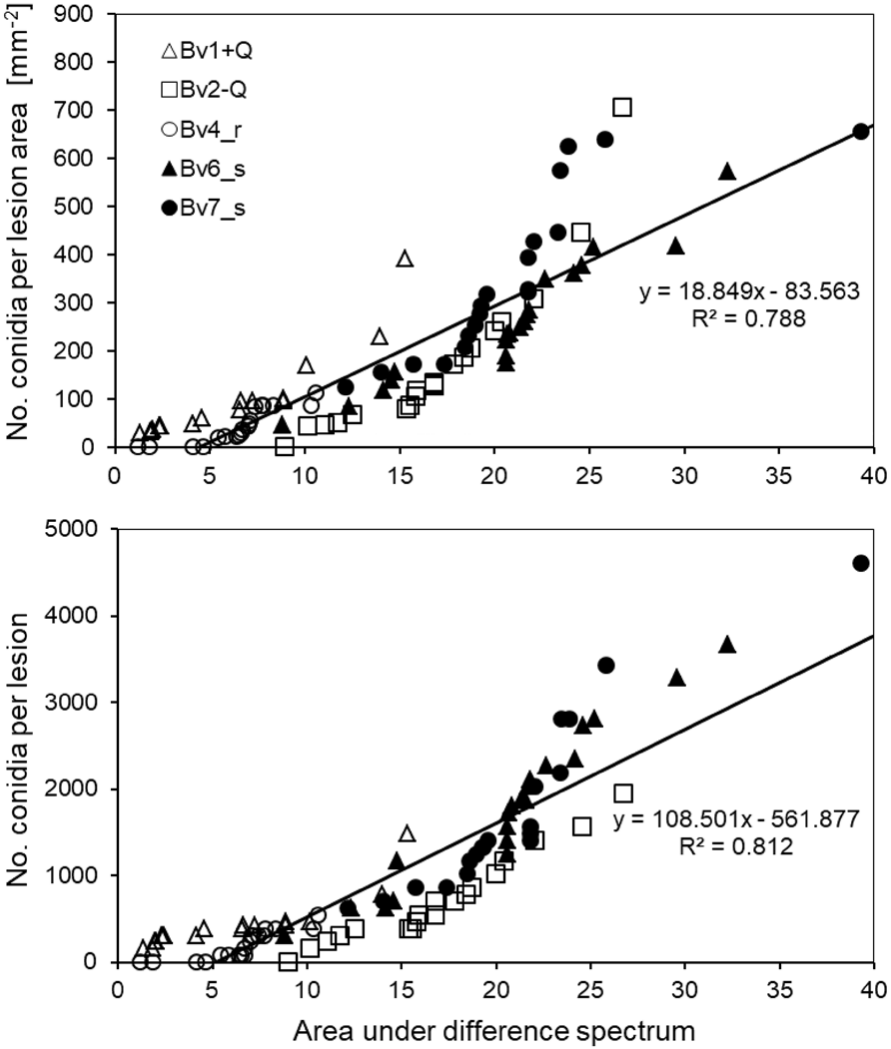
Overall relationship between the area under difference spectrum (AUDS) and the number of *C. beticola* conidia produced in lesions of five sugar beet genotypes differing in resistance to CLS. Relationship of AUDS from hyperspectral measurements taken before (19 dpi) and with (21 dpi) sporulation induced by 100% RH for 2 days to the number of conidia per lesion area (left) and to the number of conidia per lesion (right) (n = 88). Statistics for the relationships within genotypes are given in Table 1

The area under the difference spectrum (with sporulation−before induction of sporulation) was highly correlated to the number of conidia per CLS lesion as well as to the number of conidia per lesion area (Fig. 8). For the different *B. vulgaris* genotypes, quadratic regression models were better than linear relationships; however, the slopes of linear relationships reflected differences in sporulation among genotypes quite well (Table 1).

**Table 1:** Correlation between area under difference spectrum (17–19 dpi) and sporulation parameters of *Cercospora beticola* on leaf spots on five *Beta vulgaris* genotypes (n = 16)

	No. of conidia per lesion area			No. of conidia per lesion		
	R^2^ (quadratic)	R^2^ (linear)	m	R^2^ (quadratic)	R^2^ (linear)	M
Bv1 + Q	0.946	0.828	19.5	0.865	0.736	60.9
Bv2-Q	0.979	0.834	32.5	0.973	0.932	104.4
Bv4_r	0.808	0.752	13.2	0.801	0.732	61.4
Bv6_s	0.928	0.906	22.0	0.944	0.933	155.3
Bv7_s	0.849	0.795	25.2	0.910	0.908	165.0
Overall		0.788	18.8		0.812	108.5

### Effect of conidiophores and conidia on reflectance of leaf spots

**Fig. 9:**
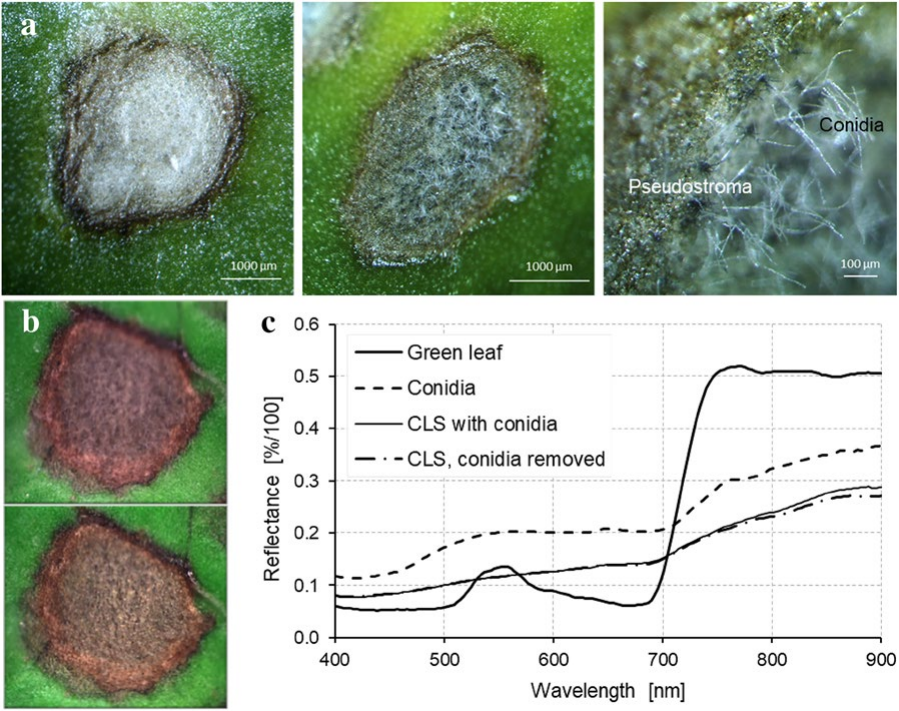
Lesion of Cercospora leaf spot (CLS) on the susceptible sugar beet genotype Bv6_s without and with conidia of *Cercospora beticola*. **a** Lesion 19 days post inoculation (dpi; left) and after incubation under 100% relative humidity for 2 days (centre and right; 21dpi). The incubation induced the formation of dark conidiophores protruding out of the leaf surface and bearing long, hyaline conidia. **b** Lesion with *C. beticola* conidia (top) and after removal of conidia by using a pencil (bottom). **c** Spectrum of lesion areas with and without conidia and spectrum of conidia taken by lateral focussing on the layer of conidia

Spectral signatures of sporulating CLS lesions on Bv6_s were analysed in more detail in order to evaluate the influence of conidia and conidiophores. Spectral reflectance was measured from the side of leaves with focus on the hyaline conidia as well as from above before and after removing the conidia (Fig. 9). The spectrum extracted from the acicular conidia was characterized by relative high reflectance in the visible range, especially from 500 to 700 nm, and an only slight increase in the near-infrared (Fig. 9c). The removal of conidia from the leaf spots had almost no influence on the reflectance from above. Apart from the near-infrared region, spectral signatures of the sporulating CLS lesions with and without spores were identical.

## Discussion

In this study, hyperspectral imaging was used to assess the sporulation of *C. beticola* on different sugar beet genotypes varying in disease susceptibility, as sporulation is a very important factor in the epidemic development of *C. beticola* in the field. In contrast to other pathogens of sugar beet leaf diseases, spore production of *C. beticola* strictly depends on the availability of high RH. With lesions fully developed, sporulation could be induced synchronously on all genotypes and the conidia produced could be harvested after a defined period of time. On susceptible plants, *C. beticola* had produced more conidia per lesion than on resistant genotypes. As sporulation rate and sporulation rate per lesion area, respectively, hardly showed a relation to lesion size, the sporulation has to be quantified directly. Although large lesions may produce high amounts of conidia, sporulation is largely confined to the central parts of CLS lesions, the size of which varies among host genotypes and even within a sugar beet leaf [21].

Moreover, differences in sporulation of *C. beticola* among host genotypes should be quantified on intact plants, since the functionality of the host metabolism seems to be essential for the full expression of both active mechanisms, resistance and susceptibility. Detached leaf assays resulted in a levelling of spore production, the variability/range of sporulation rates was higher on attached leaves. The detachment of leaves results in reduced resistance (activity) due to restricted physiological activity required for active resistance to pathogens, and higher nutrient availability for the necrotrophic pathogen compared to that in living tissue. These factors favoured sporulation of *C. beticola* on otherwise resistant genotypes of *B. vulgaris*. In susceptible genotypes, in contrast, conidiation of *C. beticola* may be affected by reduced tissue vitality required for optimum nutrition of the pathogen. The role of active processes of pathogen and host tissue in fungal sporulation is highlighted also by the significant increase in lesion size during the incubation for 2 days in all genotypes.

Leucker et al. [21] demonstrated that sugar beet genotypes with lower CLS resistance had lesions with larger centres resulting in an increased reflectance in the visible range. After induction of sporulation, the dark pigmented pseudostromata (in the host tissue) and conidiophores (arising on the leaf surface) reduced the CLS reflectance over the full range of the spectrum. In comparison, the layer of acicular, hyaline conidia above the lesion surface had only marginal effects on reflectance. The strongest changes in coloration and structure were found on susceptible genotypes. Consequently, the differences between the spectral signature before and after induction of sporulation were closely related to the amount of conidia produced per lesion. Thus, the spectral difference quantified as AUDS may be used as a proxy for fungal spore production. Hyperspectral imaging is not only able to phenotype plant characteristics, e.g. diseased leaf area, leaf area and biomass of plants, but also to quantify the activity of the pathogen depending on the compatibility of the host–pathogen interaction. It is, therefore, also very promising for the phenotyping of rather complex plant characteristics like the suitability of genotypes for pathogen sporulation.

This innovative approach is based on the hyperspectral microscope using a magnification optic compared to imaging systems on canopy or leaf level [29, 30]. Mostly, these systems were used to detect and quantify plant diseases or to characterize disease development. The approach presented here assesses spore production as an important component of quantitative plant resistance. The ability to reduce the fungal sporulation delays the disease spread and epidemic development of CLS [3, 10] and other host–pathogen systems, e.g. in peanuts resistant to *C. arachidicola* and in barley cultivars resistant to powdery mildew ( [31, 32]. In a host genotype which limits the pathogen’s sporulation rate to 50% of a susceptible host plant, the disease increase after three spore generations is only 12.5% of the increase on the susceptible host genotype. As selection for CLS resistance is mainly based on the visual assessment of the percentage of diseased leaf area of sugar beet in field trails, a closer view on the sporulation may be valuable for finding small positive effects [33]. To incorporate the quantification of the spore production into the breeding programs, a reliable and effective assessment method is necessary. However, the measurement of spores is laborious and so it has not been used routinely as a selection method in sugar beet breeding. Molecular bioassays which quantify fungal biomass, e.g. described by De Coninck et al. [22] would fail to distinguish between mycelium and spores. Findings of this study suggest that a procedure based on hyperspectral imaging may be used for the quantification of sporulation of fungal pathogens. The results from controlled conditions should help to explain differences in CLS field resistance of sugar beet genotypes which are usually assessed late in the growth period when the cumulative effects of penetration resistance, colonization resistance and sporulation resistance result in differences in disease severity.

Leucker et al*.* [21] demonstrated the suitability of the SAM algorithm for supervised classification of CLS lesion areas, i.e. margin, intermediate area, lesion centre. SAM classification of sporulating and non-sporulating lesion areas was also possible, however, the impact of cultivar-specific reflectance made it necessary to use genotype-specific reference spectra. Moreover, the formation of darkly pigmented pseudostromata in the sub-stomatal cavities may result in reduced spectral reflectance of lesion centres even before the induction of conidia formation. In this case, the stability of SAM to effects of light intensity becomes a disadvantage for the quantification of spore production. The main overall problem of the use of general reference spectra in the supervised classification of sporulation structures, however, are spectral differences among host plant genotypes which have been described earlier in sugar beet and grapevine [21, 34]. Since a step of manual processing (of lesion areas) is included, the calculation of reflectance differences (AUDS) is still time-consuming, however, further advancements in data processing may allow also for an automated assessment of sporulation. A further advantage is that AUDS calculation includes information from both, the (intensity of) pseudostromata formation and the area of sporulation.

The detailed analysis of the spectra of non-sporulating and sporulating lesion parts and conidia themselves proved that the darkly pigmented pseudostromata along with the conidiophores result in the spectral modification suitable for quantifying the sporulation of *C. beticola*—not the hyaline conidia which make only a small contribution to the overall spectral reflectance. Removal of produced conidia with droplets of water amended with surfactants may be more efficient than the mechanical approach used in this study, but causes wet surfaces and/or the uptake of water into plant tissue resulting in disturbing optical and structural effects on reflectance spectra. Since the rather erect, acicular conidia of *C. beticola* have another focus level than the leaf tissue, it seems to be possible to differentiate them spectrally from plant tissue and fungal pseudostromata. Nevertheless, the hyaline layer is rather unspecific and may be confused with effects of early powdery mildew on reflectance described by Mahlein et al. [20].

*Cercospora beticola* produces needle-like hyaline conidia on melanized conidiophores emerging through stomata or cracks of the plant surface from dark pseudostromata formed in the substomatal cavities within infected necrotic leaf tissue [14]. The differences in pigmentation between grey-brownish lesions, dark conidiophores emerging from stomata and hyaline conidia facilitates the identification of sporulating leaf spots, however, the minimal contribution of conidia to lesion spectra hampers the quantification of sporulation. The relation between genotypic resistance to *C. beticola* and the sporulating area, largely limited to the centre of CLS lesions allows the quantification of sporulation, at least of the conidiation after the first induction of spore production by suitable environmental conditions.

The relationship between *C. beticola* conidiation and AUDS was linear across all sugar beet cultivars; nevertheless, the relationship within genotypes could be described better by quadratic regressions. The cup-shaped regression curves, i.e. a kind of lag phase followed by an increasing slope may be explained by limitation of the sporulating area, i.e. the central part of CLS symptoms. Low sporulation depends on the formation of some dark-pigmented pseudostromata, increased sporulation is associated with increasing density of pseudostromata the number of which is limited due to space. Highest sporulation depends on the production of many hyaline conidia on the pseudostromata resulting in a very limited increase in AUDS.

Hyperspectral imaging proved suitable for the phenotyping of the sporulation resistance of sugar beet genotypes to *C. beticola* under controlled conditions. For the quantification of sporulation of other necrotroph pathogens, the area of leaves (or lesions) covered by asexual, darkly pigmented fruiting bodies, e.g. pycnidia and acervuli may be assessed, e.g. for *Zymoseptoria tritici*. Bousset et al. [35] estimated the density of *Leptosphaeria maculans* pycnidia produced on stem pieces from standardized RGB images. The assessment of sporulation of leaf spot pathogens with conidiophore production dispersed over typical lesions depends on the pigmentation of conidia and conidiophores (e.g. conidia and conidiophores of *Drechslera* species on leaves of barley, wheat and maize, are darkly pigmented). Sporulation of biotroph pathogens is often associated with the occurrence of disease symptoms as the mycelium and spores of the pathogen causes the typical appearance. Quantification of sporulation by calculating the diseased (= sporulating) area may be hampered by differences in the sporulation rate and the lack of spectral differences between hyphae and spores, however, differences in conidia density (of *Erysiphe betae*) may be detected by spectral differences (Mahlein and Oerke, unpublished). The potential of this technology for phenotyping even rather complex plant traits has to be elucidated in more depth also in other host–pathogen interactions.

## Conclusions

Hyperspectral imaging proved to be suitable for the quantification of fungal sporulation on leaf spots depending on the compatibility between *C. beticola* and sugar beet genotypes. The combination of spectral sensor and adequate data processing algorithm has a high potential to contribute to an automation of the quantification of fungal sporulation. The assessment of this component of partial resistance to plant pathogens may improve and accelerate the phenotyping procedure in breeding of crops for disease resistance under controlled conditions, at least in suitable host–pathogen systems.

## Supplementary information

**Additional file 1: Fig. S1.** Correlation between size of Cercospora leaf spots and the number of *C. beticola* conidia per lesion and the number of conidia per lesion area, respectively, produced within 2 days of incubation under 100% relative humidity.
