# Supplementary material for: Sensory assessment of Cercospora beticola sporulation for phenotyping the partial disease resistance of sugar beet genotypes
Source: Plant Methods. 2019 Nov 16;15:133. doi: 10.1186/s13007-019-0521-x (PMC6858659; doi:10.1186/s13007-019-0521-x)

Additional file 1: Figure S1. Correlation between size of Cercospora leaf spots and the number of *C. beticola* conidia per lesion and the number of conidia per lesion area, respectively, produced within 2 days of incubation under 100% relative humidity.


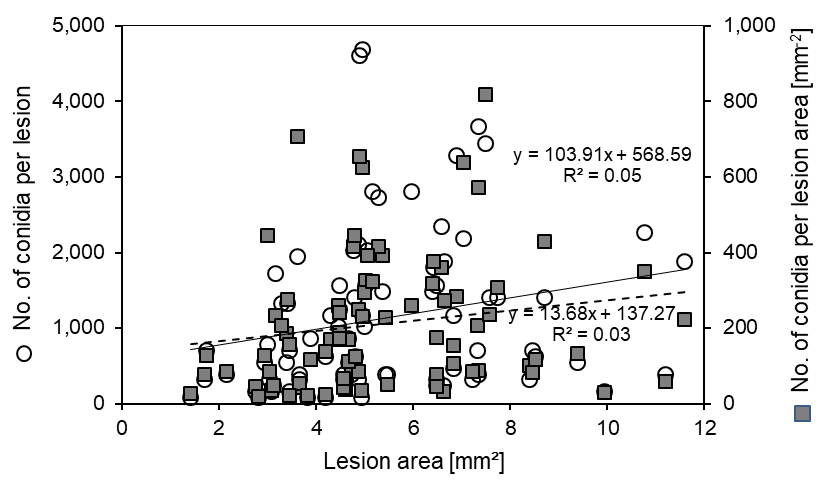

Supplement: Supplementary file 1 — Additional file 1: Fig. S1. Correlation between size of Cercospora leaf spots and the number of C. beticola conidia per lesion and the number of conidia per lesion area, respectively, produced within 2 days of incubation under 100% relative humidity. [file 13007_2019_521_MOESM1_ESM.docx]
